# Severe acute respiratory syndrome coronavirus 2 can be detected in exhaled aerosol sampled during a few minutes of breathing or coughing

**DOI:** 10.1111/irv.12964

**Published:** 2022-01-17

**Authors:** Emilia Viklund, Spela Kokelj, Per Larsson, Rickard Nordén, Maria Andersson, Olof Beck, Johan Westin, Anna‐Carin Olin

**Affiliations:** ^1^ Occupational and Environmental Medicine, School of Public Health and Community Medicine, Institute of Medicine, Sahlgrenska Academy University of Gothenburg Gothenburg Sweden; ^2^ Department of Infectious Diseases, Institute of Biomedicine, Sahlgrenska Academy University of Gothenburg Gothenburg Sweden; ^3^ Department of Clinical Microbiology Sahlgrenska University Hospital Gothenburg Sweden; ^4^ Department of Clinical Neuroscience Karolinska Institute Solna Sweden

**Keywords:** aerosol, breath test, COVID 19, respiratory aerosols, SARS‐CoV‐2

## Abstract

**Background:**

The knowledge on the concentration of viral particles in exhaled breath is limited. The aim of this study was to explore if severe acute respiratory syndrome coronavirus 2 (SARS‐CoV‐2) can be detected in aerosol from subjects with the coronavirus disease 2019 (COVID‐19) during various types of breathing and coughing and how infection with SARS‐CoV‐2 may influence the number and size of exhaled aerosol particles.

**Methods:**

We counted and collected endogenous particles in exhaled breath in subjects with COVID‐19 disease by two different impaction‐based methods, during 20 normal breaths, 10 airway opening breaths, and three coughs, respectively. Breath samples were analyzed with reverse transcription real‐time polymerase chain reaction (RT‐PCR).

**Results:**

Detection of RNA in aerosol was possible in 10 out of 25 subjects. Presence of virus RNA in aerosol was mainly found in cough samples (*n* = 8), but also in airway opening breaths (*n* = 3) and in normal breaths (*n* = 4), with no overlap between the methods. No association between viral load in aerosol and number exhaled particles <5 μm was found. Subjects with COVID‐19 exhaled less particles than healthy controls during normal breathing and airway opening breaths (all *P* < 0.05), but not during cough.

**Conclusion:**

SARS‐CoV‐2 RNA can be detected in exhaled aerosol, sampled during a limited number of breathing and coughing procedures. Detection in aerosol seemed independent of viral load in the upper airway swab as well as of the exhaled number of particles. The infectious potential of the amount of virus detected in aerosol needs to be further explored.

## 1 INTRODUCTION

The coronavirus disease 2019 (COVID‐19), caused by the severe acute respiratory syndrome coronavirus 2 (SARS‐CoV‐2), is assumed to mainly be transmitted by respiratory droplets. However, probable aerosol transmission has been reported to occur under certain conditions.^1^, ^2^ The knowledge on the concentration of SARS‐CoV‐2 particles (viral load) in exhaled breath samples is limited as well as the size and concentration of exhaled endogenously generated droplets in relation to viral load. Moreover, the relation between the viral load in upper airway diagnostic samples and aerosol samples needs further clarification.

An aerosol contains micro‐sized particles generated from the respiratory tract lining fluid (RTLF) in the airways.^3^ The smallest fraction exhaled, that is, particles <5 μm, are mainly formed on inhalation when bronchiolar fluid film bursts.^4^, ^5^ Particle formation in small airways, occurring at normal breathing, can be increased by deep exhalation followed by deep inhalation resulting in airway closure and re‐opening.^5^, ^6^ The individual number and size of exhaled particles differ substantially both between and within one subject and may depend on several factors, for instance, the precise breathing pattern used.^4^, ^7^, ^8^

It has been suggested that viruses may be enriched in exhaled particles with a diameter of <5 μm.^9^ This is supported by recent findings indicating higher SARS‐CoV‐2 viral load in exhaled particles <5 μm compared with those >5 μm during speech and song from COVID‐19 patients^10^ as well as in breath samples from anesthetized SARS‐CoV‐2‐infected macaques.^11^

The PExA (particles in exhaled air) method is optimized for collection of small airway particles with a size range of approximately 0.4–5 μm, using the airway opening maneuver.^12^ Another aerosol‐collection device; BE (Breath Explor), also based on impaction but without particle counting and airflow data, has recently been developed.^13^ Due to the design of BE where sample is collected immediately at the mouth opening, it seems highly plausible that also particles >5 μm can be sampled using this device.

The main objective of this study was to explore if SARS‐CoV‐2 can be detected in aerosol from subjects newly diagnosed with COVID‐19, using PExA and BE. If so, we aimed to compare the viral load in exhaled aerosol collected during various types of breathing and coughing and to explore how infection with SARS‐CoV‐2 may influence the number and size of exhaled aerosol particles <5 μm.

## 2 MATERIALS AND METHODS

### 2.1 Study design and study subjects

Subjects with mild symptomatic COVID‐19 disease were recruited for a first substudy (i) during September–October 2020. A second substudy (ii) took place from April to May 2021. Both studies were carried out at Sahlgrenska University Hospital, Gothenburg, Sweden. Eligible subjects were hospital health care workers testing positive for COVID‐19 according to the routine testing of hospital staff currently used at the time for the study inclusion. In the first substudy (i), diagnosis was confirmed by positive reverse transcription real‐time polymerase chain reaction (RT‐PCR) analysis of a combined oropharyngeal and nasopharyngeal (oro/nasopharyngeal) swab sample, and in the second substudy (ii) by a positive Rapid COVID‐19 Antigen Test (CLINITEST®) (Siemens, Healthineers, Erlangen, Germany) obtained by nasopharyngeal swab sampling.

Breath sampling was performed (i) several days after a positive PCR of oro/nasopharyngeal sample, and (ii) immediately after antigen testing at the staff testing station. A positive antigen test was confirmed with PCR analysis of an oro/nasopharyngeal swab sample, taken the same day. All control subjects were recruited among hospital health care workers without symptoms of COVID‐19 and performed a Rapid SARS‐CoV‐2 Antigen Test Card (Boson Biotech) (Xiamen Boson Biotech Co., Ltd, Xiamen, China) to confirm their negative COVID‐19 status at the time of the breath sampling.

### 2.2 Breath sampling by PExA

**FIGURE 1:**
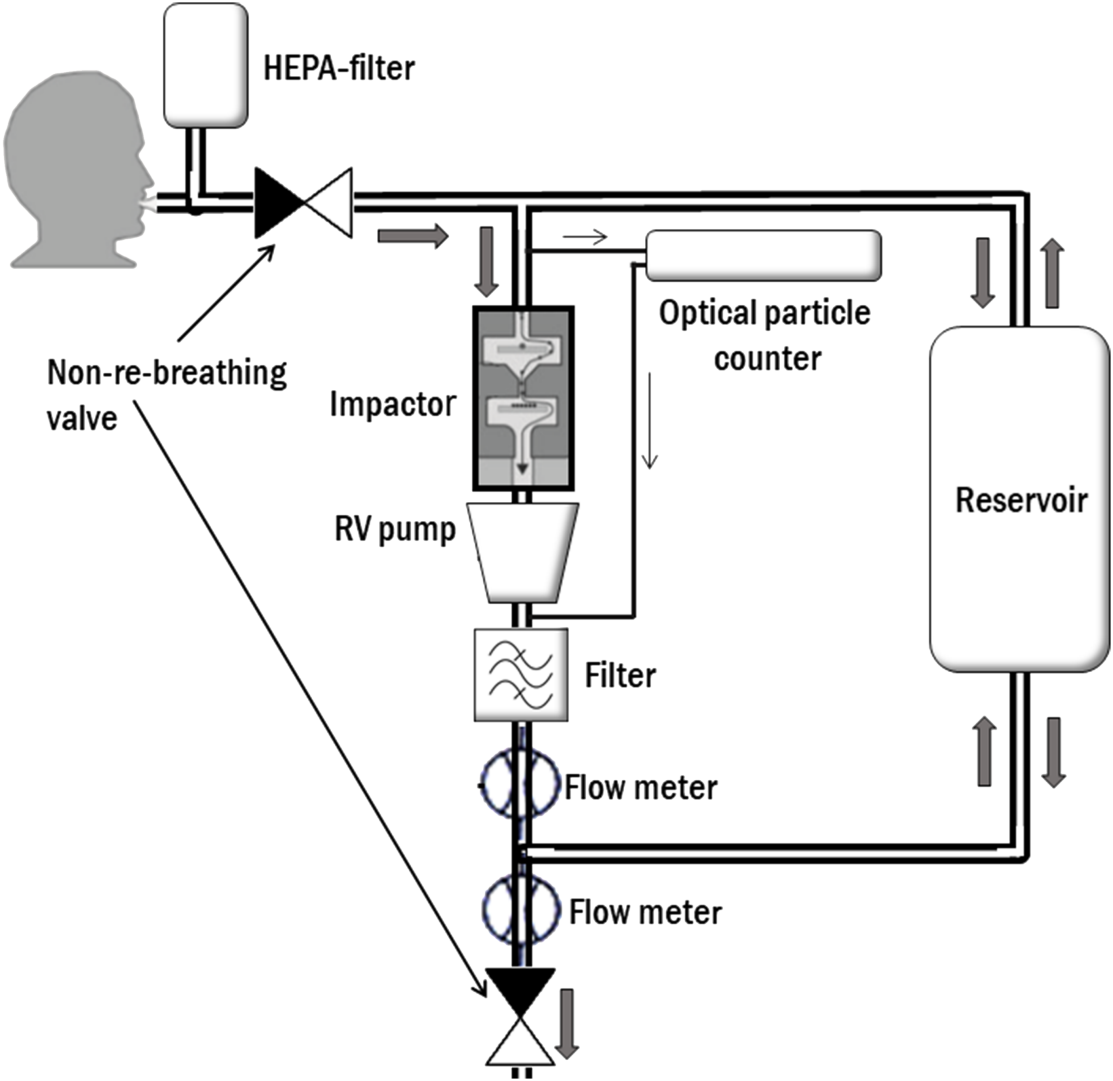
Schematic illustration of the particles in exhaled air (PExA) instrument set‐up at collection. Subject breaths through a mouthpiece, connected to a two‐way, non‐re‐breathing valve, where inhalation goes through a high‐efficiency particle arresting (HEPA) filter and exhalation goes into the instrument. An optical particle counter samples a fraction of the exhaled air with a constant flow of 20 ml/s. The two stage inertial impactor collects particles according to size by the control of a rotary vane (RV) pump with a constant flow of 230 ml/s. A reservoir handles exhalations that exceeds the flow rate through the impactor

Characterization and collection of PExA (Figure 1) was made using the PExA instrument (PExA AB, Gothenburg, Sweden). The principles of the PExA method have been described in detail previously by Almstrand et al.^12^ In brief, the PExA instrument uses an optical particle counter (Grimm 1.108, Grimm Aerosol Technik GmbH, Ainring, Germany) and a cascade impactor for particle collection. The particle counter measures particle number concentrations and particle size in eight size bins covering the particle range of approximately 0.4–5 μm in diameter. The PExA instrument has two collection plates covered with thin sampling membranes of hydrophilic polytetrafluorethylene (PTFE) (FHLC02500, Millipore, Billerica, MA, USA). Particles with an aerodynamic diameter above approximately 5 μm reaching the instrument were collected on the upper plate, and particles between 0.4 and 5 μm in diameter were counted and collected on the lower plate in the impactor.

Subjects wore a nose clip and inhaled air through a high‐efficiency particle arresting (HEPA) filter to remove ambient particles so that only endogenous particles were exhaled into the PExA instrument.

The separate breathing procedures used for particle collection were as follows:

- *Normal breathing*: relaxed breathing for 20 breaths.
- *Airway opening maneuver*: Deep exhalation to residual volume (RV), 5‐s breath hold at RV followed by a rapid inhalation to total lung capacity (TLC) and finally a relaxed exhalation to RV. Repeated 10 times.
- *Coughs*: Deep inspiration to TLC followed by a cough. Repeated three times.

The PTFE membranes on each impactor plate with sampled particles was transferred to separate, 1.5‐ml SC microtube PC‐PT cryotubes (Sarsteds, Nümbrecht, Germany) and stored at −80°C, prior to RNA extraction. Particle number concentrations are expressed as *n* * 1,000 (kn).

### 2.3 Breath sampling with BE

The handheld BE instrument (Munkplast AB, Uppsala, Sweden) was used for collection of particles in aerosol, without counting and size fractioning. The principles of the BE method have been described in detail previously by Seferaj et al.^13^ Sampling was performed according to instructions by the manufacturer (VER: 2019‐05‐06), with minor modifications. Subjects wore a nose clip throughout the collection and performed 20 normal relaxed exhalations into the device.

Samples were then stored at −80°C, prior to RNA extraction.

### 2.4 RNA extraction and detection of SARS‐CoV‐2 in exhaled particles

The SARS‐CoV‐2 PCR analysis, from which the cycle threshold (Ct) values were obtained, was performed in a Cobas® 6800 (Roche Diagnostics, Mannheim, Germany) according to manufacturer's instructions at the Virology unit at the department of Clinical Microbiology, Sahlgrenska University Hospital.

PTFE membranes were incubated overnight in 2 ml of lysis buffer, and this volume was used for extraction of total nucleic acid in an EasyMag instrument (Biomerieux, Marcy l'Étoile, France) eluted in 110 μl. BE membranes were thoroughly washed with a volume of 1.2 ml of buffered sodium hydroxide by pipetting and vortexing, and 1‐ml volume was used for nucleic acid extraction by a MagNA Pure LC instrument (Roche Diagnostics, Mannheim, Germany) using the Large Volume Total Nucleic Acid isolation kit. The total nucleic acid was eluted in 50‐μl volume.

Real‐time PCR was performed in duplicates in a QuantStudio 6 (Applied Biosystems, Foster City, United States) instrument by using a 50‐μl reaction volume including 10 μl of purified nucleic acid, primers, and probe targeting SARS‐CoV‐2 polymerase gene (RdRP)^14^ and Taqman Fast Virus 1‐step Mastermix (Applied Biosystems). After a reverse transcription step at 46°C for 30 min followed by 10 min of denaturation at 95°C, 45 cycles of two‐step PCR was preformed (15 s at 95°C, 60 s at 58°C).

Viral load refers to Ct values, a proxy for inverse viral concentration of detected SARS‐CoV‐2 RNA. All samples only positive for SARS‐CoV‐2 in one reaction with Ct > 35 were reanalyzed to confirm the result.

### 2.5 Statistical analysis

Statistical analyses were performed using the IBM SPSS software, version 26.0 (SPSS, Chicago, IL). Nonparametric tests were used due to few subjects and skewed distributions. Mann–Whitney *U* test was used for comparison of continuous data and chi‐square tests using Fisher's exact significance (two‐sided) for comparison of categorical data between subjects with COVID‐19, with and without detectable SARS‐CoV‐2 RNA.

## 3 RESULTS

Ten subjects with confirmed COVID‐19 were included in substudy (i), where aerosol sampling took place 8 (5–11) days after symptom onset and 3 (1–7) days after PCR‐positive oro/nasopharyngeal swab. A positive aerosol sample, collected with PExA during coughing, was found in the one subject with the shortest symptom duration (5 days). On the basis of this finding, we then re‐designed the protocol in order to minimize the time between symptom onset and aerosol sampling.

Twenty‐five subjects with COVID‐19 and with a symptom duration of 2 (0–9) days, and 11 controls, were included in the second substudy (ii), where aerosol was sampled the same day as confirmed COVID‐19. Aerosol sampling time varied depending on breathing pattern; approximately 1 min for three coughs, 2 min for 20 normal breaths, and 5 min for 10 airway opening maneuvers. PCR findings and number of exhaled particles from subjects with COVID‐19 in substudy (ii), sampled from different breathing maneuvers by the use of PExA and BE, are presented in Table 1. Nine out of 25 subjects with COVID‐19 had detectable SARS‐CoV‐2 RNA in any PExA sample and two out of 25 subjects in BE samples, resulting in 10 aerosol positive subjects in total. One of the subjects were positive for SARS‐CoV‐2 with both PExA and BE, however, using different breathing maneuvers. The two subjects with positive antigen test but negative oro/nasopharyngeal swab PCR both had detectable RNA in aerosol, one in normal breathing and one in cough. Overall, viral load in oro/nasopharyngeal samples was high in comparison with that of samples from aerosol, and no association between viral load in upper airways and aerosol specimens was found.

**TABLE 1:** Polymerase chain reaction (PCR) results for oro/nasopharyngeal and aerosol samples, and number of exhaled particles for aerosol samples, sampled during different breathing procedures, from 25 subjects with coronavirus disease 2019 (COVID‐19)

	Clinical test	PExA (<5 μm)						BE
	Oro/nasoparyngeal swab	Normal breaths		Airway opening maneuvers		Coughs		Normal breaths
Subject ID	Ct value	Ct value	PEx/breath	Ct value	PEx/breath	Ct value	PEx/breath	Ct value
1	17.2	33.8	0.0	—	44.9	35.4	3.5	—
2	17.4	—	md	33.4	47.3	36.2	10.1	37.7
3	17.5	—	0.1	—	16.9	—	7.3	—
4	18	—	0.0	—	42.0	36.5	2.9	—
5	18.4	—	0.1	31.8	25.0	34.5	5.5	—
6	19.1	—	0.8	—	104.9	—	10.4	—
7	19.1	—	0.1	—	17.8	—	0.9	—
8	19.5	—	0.1	—	30.2	—	6.5	37.5
9	19.6	—	0.1	—	28.5	—	2.0	—
10	19.9	—	0.2	—	21.1	—	17.7	—
11	20.1	—	0.0	—	28.0	—	1.7	—
12	20.3	—	0.2	—	18.6	—	1.6	—
13	20.3	—	0.0	—	5.6	—	1.4	—
14	20.5	—	0.1	—	41.4	29.5	217.4	—
15	20.8	—	0.0	—	6.2	—	2.9	—
16	22.1	—	0.7	36.8	42.0	36.4	7.2	—
17	22.7	—	0.0	—	5.0	—	1.1	—
18	23.4	—	0.0	—	47.7	—	2.3	—
19	23.5	—	0.0	—	1.9	—	0.9	—
20	24.7	—	0.1	—	6.0	—	20.9	—
21	25.3	—	0.0	—	67.6	35	9.5	—
22	25.5	—	0.0	—	82.4	—	13.2	—
23	26.4	—	0.2	—	15.5	—	20.9	—
24	—	—	0.1	—	29.5	35.8	94.8	—
25*	—	36.8	0.1	—	86.7	—	57.5	—

*Note*: Aerosol sampling with PExA was performed during 20 normal breaths, 10 airway opening maneuvers, and three coughs, respectively. Aerosol sampling with BE was performed during 20 normal breaths. Exhaled particles (PEx) are expressed as *n* * 1,000 (kn) per breath. Subjects are numbered according to rising Ct‐value from oro/nasopharygeal swab.

Abbreviations: Ct; cycle threshold; md; missing data (number of exhaled particles failed to be registered).

* ID 25 also had a positive PCR in sample of particles >5 μm (Ct value = 37), collected with PExA at tidal breathing.

Table 2 displays demographical data for subjects with versus without detected SARS‐CoV‐2 RNA in any aerosol sample, as well as healthy control subjects. Age distribution, symptom pattern, and symptom duration were similar among subjects with and without detectable SARS‐CoV‐2 RNA in the aerosol, apart from nasal congestion that was more common among those with RNA detected only in upper airway swab and not in aerosol.

**TABLE 2:** Clinical and demographic characteristics of 25 subjects with coronavirus disease 2019 (COVID‐19) and 11 healthy control subjects, based on positive or negative severe acute respiratory syndrome coronavirus 2 (SARS‐CoV‐2) RNA findings in aerosol collected with particles in exhaled air (PExA) instrument

Variable	Pos aerosol PCR (*n* = 9)	Neg aerosol PCR (*n* = 16)	Controls (*n* = 11)	*P* value
Females	5 (56)	12 (75)	7 (64)	0.593
Age, years	48 (33–61)	42 (23–58)	42 (23–67)	0.463
Current smoker	0	1 (7)	1 (9)	
Symptom duration, days	2 (1–9)	2 (0–9)	0 (0)	0.834^a^
Symptoms				
Shortness of breath at rest	1 (11)	2 (13)	0 (0)	1.000^a^
Cough	8 (89)	9 (56)	0 (0)	0.182^a^
Fever	5 (56)	5 (31)	0 (0)	0.397^a^
Runny nose	7 (67)	11 (69)	0 (0)	1.000^a^
Nasal congestion	2 (22)	11 (69)	0 (0)	0.017^a^
Sore throat	6 (67)	7 (44)	0 (0)	0.411^a^
Swab, Ct value from RT‐PCR	18.4 (17.2–25.3)	20.3 (17.5–26.4)	—	0.175^a^

*Note*: Data are presented as median (min–max) or *n* (%). Chi‐square tests using Fisher's exact significance (two‐sided) for categorical data. Mann–Whitney *U* test for continuous data.

Abbreviation: RT‐PCR, reverse transcription real‐time polymerase chain reaction; Ct; cycle threshold.

^a^ Only tested between pos aerosol PCR and negative aerosol PCR.

**FIGURE 2:**
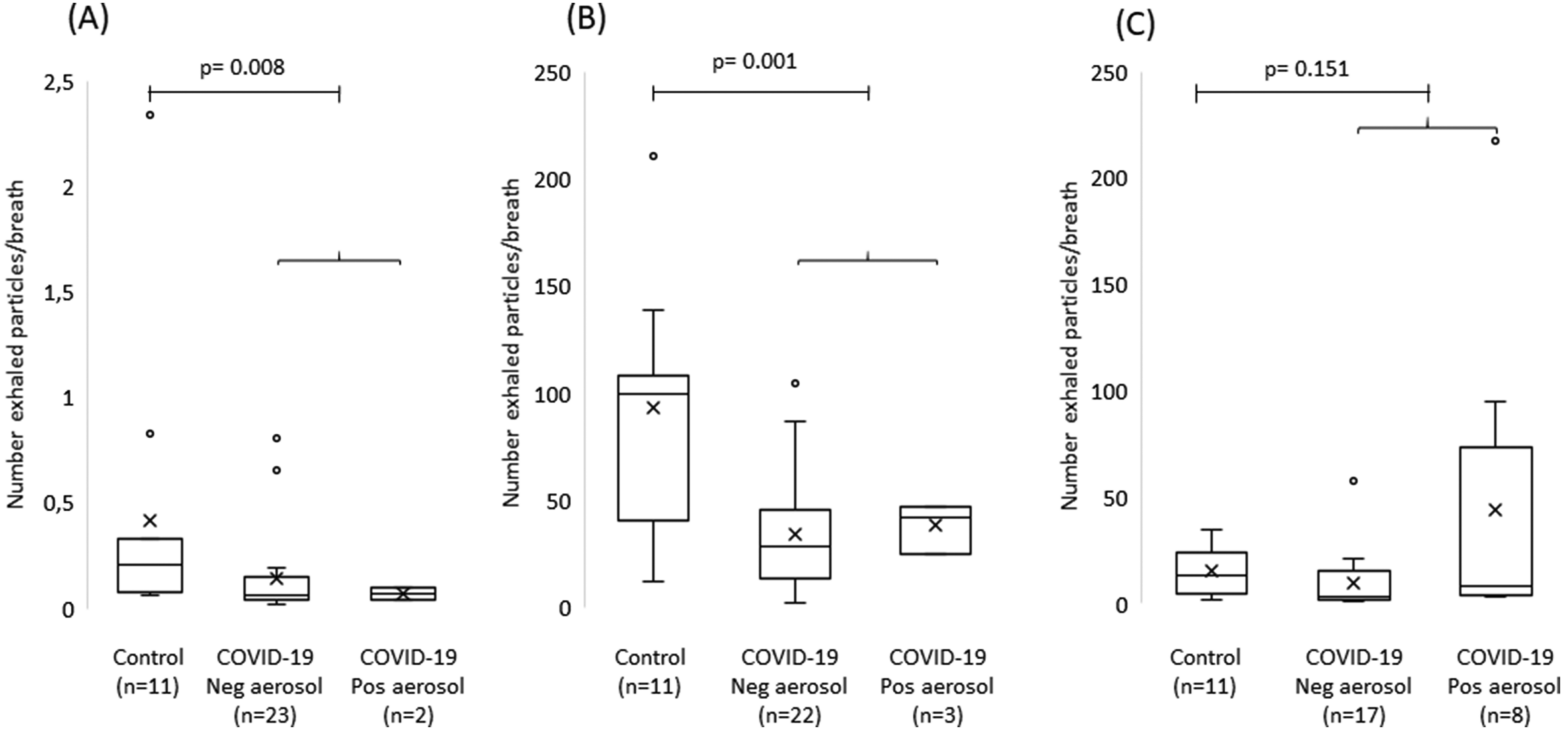
Box plots of particle number concentrations among control subjects (*n* = 11) and among coronavirus disease 2019 (COVID‐19) subjects (*n* = 25) with and without detectable severe acute respiratory syndrome coronavirus 2 (SARS‐CoV‐2), sampled during (A) normal breathing, (B) airway opening maneuver, and during (C) coughs. Particle number concentrations expressed as *n* * 1,000 (kn) per breath. Horizontal lines represent the median, cross represents the mean, boxes represent the interquartile range, and whiskers represent the range. Circles represent subjects and circles outside box represent subjects with extreme values. *P* values refers to Mann–Whitney *U* test between control subjects and all subjects with COVID‐19

Ten airway opening maneuvers produced a hundred times more particles per exhalation than that of normal breaths, as illustrated in Figure 2. Subjects with COVID‐19 exhaled less particles during normal breathing and airway opening maneuver as compared with controls.

**FIGURE 3:**
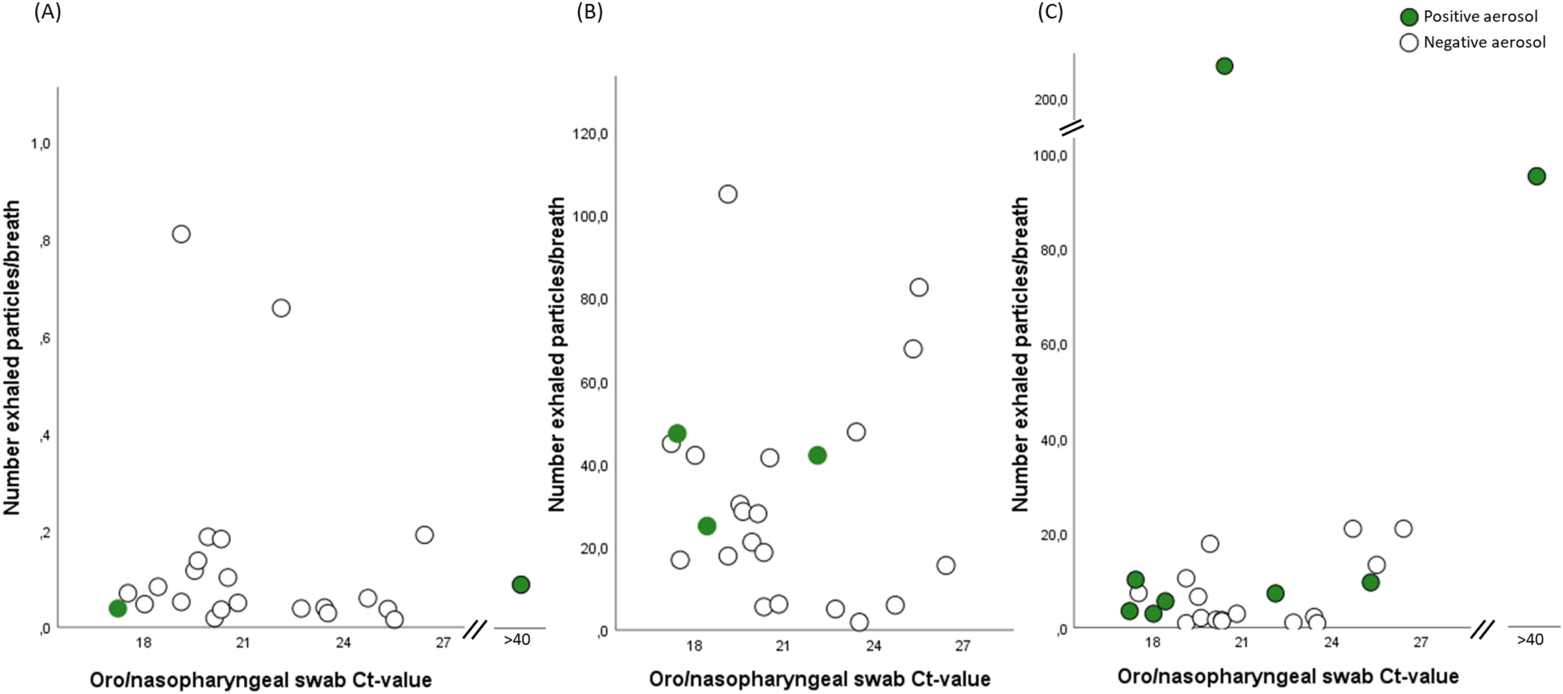
Number of exhaled particles/breath collected at (A) normal breathing, (B) airway opening and at (C) cough, in comparison with viral load in oro/nasopharyngeal swab samples, in subjects with coronavirus disease 2019 (COVID‐19) (*n* = 25). Number of exhaled particles are expressed as *n* * 1,000 (kn) per breath. Subjects with COVID‐19 and with a positive aerosol sample but with a negative reverse transcription real‐time polymerase chain reaction (RT‐PCR) from oro/nasopharyngeal swab samples are here presented with a cycle threshold (Ct) value above 40. Particle data are missing in one case in (A)

The number of exhaled particles was neither associated with viral load in oro/nasopharyngeal swab samples nor with a positive aerosol sample in any of the breathing procedures studied, apart from two aerosol positive subjects at cough with extreme values of number exhaled particles; see Figure 3.

**FIGURE 4:**
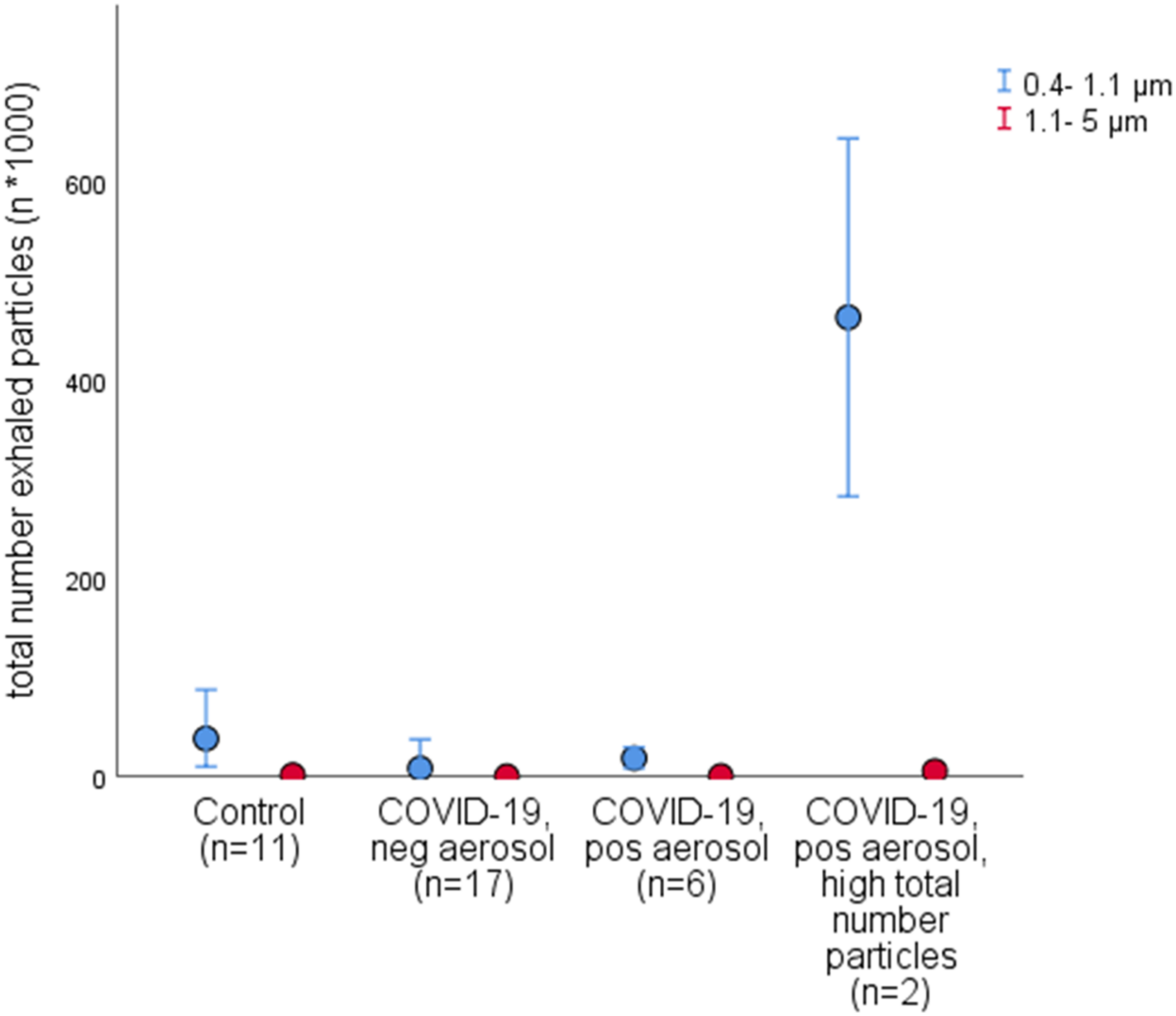
Total number of exhaled particles <5 μm, in two size intervals, sampled from cough, in control subjects (*n* = 11) and in subjects with coronavirus disease 2019 (COVID‐19) (*n* = 25) subdivided based on negative or positive severe acute respiratory syndrome coronavirus 2 (SARS‐CoV‐2) aerosol and with positive aerosol further subdivided into two groups with extreme values of total number exhaled particles in a separate group. Number of exhaled particles are expressed as *n* * 1,000 (kn). The results from the measured particle number concentrations in eight size intervals, covering a range of 0.4–5 μm, have been merged into two size intervals (0.4–1.1 μm and 1.1–5 μm, respectively). Dots represent the median; error bars represent the 95% confidence interval (CI)

Size distribution of exhaled particles <5 μm at cough showed number particles between 0.4 and 1.1 μm to be markedly higher in the two subjects with positive aerosol and highest total number exhaled particles at cough, in comparison with the rest of the study subjects at cough, as seen in Figure 4.

## 4 DISCUSSION AND CONCLUSION

The present study shows that SARS‐CoV‐2 RNA can be detected in exhaled particles of diameter size <5 μm, in some but not all subjects with COVID‐19 early in the course of the disease, despite sampling a small amount of aerosol particles. Subjects with COVID‐19 exhaled lower number of particles <5 μm during normal breathing but also following airway opening maneuver, than healthy controls, indicating an effect of the disease on particle formation. No obvious associations were found neither between viral load in oro/nasopharyngeal samples and PExA or BE aerosol samples nor between the number of particles exhaled and the detection of SARS‐CoV‐2 RNA in aerosol.

SARS‐CoV‐2 RNA was detectable in aerosol samples from 40% of subjects with COVID‐19; PExA and BE results merged. This is somewhat lower than recent finding in aerosol from COVID‐19 patients, where 59% of subjects had detectable RNA,^10^ however, using longer sampling time (15–30 min) in comparison with our study (1–5 min). Our findings highlight the very small amount of aerosol required in order to detect SARS‐CoV‐2 RNA under certain conditions.

The possibility to detect virus in exhaled breath likely depends on the method used for sampling, the amount of virus on the surface where aerosol particles are formed, as well as the breathing pattern. Aerosol sampling by impaction has been used also in previous studies.^10^, ^11^ The novelty in the present study is counting and size fractioning of particles between 0.4 and approximately 5 μm, and the use of airway opening maneuver as one of the breathing patterns studied.

Presence of virus RNA in aerosol detected with PExA was almost exclusively found on the impactor stage sampling particles <5 μm, in comparison with >5 μm, in line with previous findings^10^, ^11^ and further supported by a recent review suggesting that SARS‐CoV‐2 RNA is enriched in aerosol with particle sizes <5 μm.^9^

Number of exhaled particles <5 μm varied greatly depending on the breathing pattern applied, in line with other studies.^4^, ^5^, ^15^ Cough, with the largest number of positive aerosol samples (*n* = 8), has been suggested to generate particles from the upper airways as a consequence of shear stress and high exhalation flows^15^, ^16^, ^17^ and from vibrating the vocal chords.^4^ Normal breathing and airway opening maneuver on the other hand has been suggested to generate particles mainly from the small airways through film bursting of RTLF,^5^, ^6^, ^16^ with an amplified particle formation during airway opening maneuver. In the present study, there was however neither any overlap between subjects with positive aerosol during normal breathing and airway opening maneuver using the PExA method nor between PExA and BE samples from 20 normal breaths. The low amount of sampled aerosol particles at normal breathing might potentially explain our low detection rate.

SARS‐CoV‐2 has been shown to be present both in upper, central, and lower airways^18^ but may exert most of its deleterious effects in the terminal bronchioles and alveoli. Positive aerosol findings in airway opening maneuver samples may therefore indicate that dissemination of virus to the distal airways occur early in the disease. However, we cannot fully exclude contamination from upper airways during the airway opening maneuver, but it is unlikely.

Positive aerosol samples were not associated with number of exhaled particles, apart from two positive aerosol samples at cough with extreme values of exhaled particles. The 100‐fold lower number exhaled particles at normal breathing in comparison to cough and airway opening, but still with two positive aerosol samples, further highlight this lack of association. Regardless, aerosol samples were extracted from particles <5 μm and collected during only a few minutes, meaning that an aerosol transmission of SARS‐CoV‐2 may occur during a short amount of time of breathing, coughing or airway opening.

The viral load in all aerosol samples was low in comparison with the oro/nasopharyngeal samples, which contain a larger volume of secretion, and is in line with previous studies.^10^, ^19^, ^20^ Important to note, detection of RNA is not equal to detection of contagious virus. Neither do we know under which conditions (e.g., distance or duration) the small amount of virus detected in our aerosol samples is enough to infect a susceptible individual, nor can we confirm the viability of aerosolized virus, due to lack of cell culture data. A study on influenza virus reported the infective dose required to initiate infection by inhalation of aerosols to be a hundredth times smaller than the dose required for intranasal inoculation.^21^ Nevertheless, our results support the notion that exhaled aerosol may carry virus particles that under certain conditions, might cause transmission of disease.

Markedly higher number of exhaled particles sampled during cough were generated by two subjects, where size fractioning of particles indicated strikingly higher number of particles <1.4 μm (Figure 3). It is well known that there is a large span of the number of exhaled particles^4^, ^22^, ^23^ for yet unknown reasons. Individuals producing more particles (e.g., “high producers”), as well as aerosol generating breathing patterns^1^ might be of extra interest in trying to identify “super spreaders” of viral respiratory infections. In light of this, one subject in the present study, with the highest amount of exhaled particles/breath, also had the highest viral load of all aerosol samples. On the other hand, the two subjects with detectable RNA during normal breathing, collected with PExA, generated approximately 100‐fold lower number of particles, suggesting particle formation at airway regions that contain high amount of viruses.

Exhalation following airway opening maneuver as well as normal breathing generated markedly lower numbers of exhaled particles in subjects with COVID‐19 than in healthy subjects, independent of SARS‐CoV‐2 RNA detection, for yet unknown reasons. Potentially, viral infection may change the properties of the lining fluid, for example, viscosity amount of lung lining fluid, that in turn may influence the formation of particles originating from the small airways and/or more small airways may be closed.

The major limitation with the study is the limited number of subjects included, which was due to difficulties to recruit newly infected subjects. We chose to compare viruses in the exhalate during different breathing maneuvers and with only a few breaths per maneuver, resulting in a rather small sampling volume from each individual. A larger sample volume would have been beneficial. Nevertheless, we managed to detect virus RNA in aerosol particles <5 μm from all three of the breathing patterns used, indicating a rather small amount of exhaled air needed to detect SARS‐CoV‐2 RNA. Moreover, the PExA instrument is neither designed for collections of particles from cough nor for the counting of particles >5 μm. It is also difficult to standardize coughing maneuvers. Also, potential incorrect sampling or handling of swab samples as well as of aerosol samples might have resulted in both false negative and positive results, difficult to control for, but seem not to induce any systemic error.

In conclusion, our results show that SARS‐CoV‐2 RNA can be detected in exhaled aerosol sampled during a limited number of breathing and coughing procedures, early in the disease course of COVID‐19. The two sampling methods used for detection at normal breathing did not overlap. Detection in aerosol seemed independent of viral load in the upper airway swab as well as of the exhaled number of particles. The infectious potential of the amount of virus detected in aerosol needs to be further explored.

## AUTHOR CONTRIBUTIONS

**Emilia Viklund:** Conceptualization; data curation; formal analysis; investigation; methodology; project administration. **Spela Kokelj:** Conceptualization; formal analysis; investigation; methodology; project administration. **Per Larsson:** Conceptualization; formal analysis; methodology. **Rickard Nordén:** Formal analysis; methodology; resources. **Maria Andersson:** Data curation; formal analysis; methodology; resources. **Olof Beck:** Methodology; resources. **Johan Westin:** Conceptualization; formal analysis; funding acquisition; methodology; project administration; resources; supervision. **Anna‐Carin Olin:** Conceptualization; funding acquisition; methodology; resources; supervision.

## FUNDING INFORMATION

This work was supported by Region Västra Götaland ALF research funds (grant numbers ALFGBG‐719911 to Johan Westin, ALFGBG 725071 to Anna‐Carin Olin) and by Stockholm County Council (grant number SLL ALF20190536 to Olof Beck). The funders had no role in study design, data collection and analysis, decision to publish, or preparation of the manuscript.

### PEER REVIEW

The peer review history for this article is available at https://publons.com/publon/10.1111/irv.12964.

## Data Availability

The data that support the findings of this study are available from the corresponding author upon reasonable request.
